# Supplementary material for: Mapping the evolution of fertility support policies in China: A content and instrumental analysis
Source: PLoS One. 2025 Oct 9;20(10):e0332137. doi: 10.1371/journal.pone.0332137 (PMC12510515; doi:10.1371/journal.pone.0332137)
Supplement: S1 Appendix — (ZIP) [file pone.0332137.s001.zip › S1 Appendix. 226 original policy documents/12-国家人口计生委关于全面深入实施新农村新家庭计划的意见(FBM-CLI-4-149219).doc]

国家人口计生委关于全面深入实施新农村新家庭计划的意见

**发布部门：** [国家人口和计划生育委员会(含国家计划生育委员会)(已撤销)](https://www.pkulaw.com/law/chl?Aggs.IssueDepartment=60304) [机构沿革](javascript:void(0))

**发文字号：**人口宣教〔2011〕32号

**发布日期：**2011.04.01

**实施日期：**2011.04.01

**时效性：** [现行有效](https://www.pkulaw.com/law/chl?Aggs.TimelinessDic=01)

**效力级别：** [部门工作文件](https://www.pkulaw.com/law/chl?Aggs.EffectivenessDic=XE0303)

**法规类别：** [人口计划管理](https://www.pkulaw.com/law/chl?Aggs.Category=09701)

国家人口计生委关于全面深入实施新农村新家庭计划的意见
（人口宣教〔2011〕32号）

各省、自治区、直辖市人口计生委，计划单列市、新疆生产建设兵团人口计生委：
　　为深入贯彻落实党的十七届五中全会精神，全面做好人口工作，进一步提高“十二五”时期农村人口和计划生育工作水平，现就全面深入实施新农村新家庭计划提出如下意见。
　　**一、**充分认识全面深入实施新农村新家庭计划的重要意义
　　实施新农村新家庭计划，是《中共中央[国务院关于全面加强人口和计划生育工作统筹解决人口问题的决定](https://www.pkulaw.com/chl/5e7b01010a4102ffbdfb.html)》（中发〔2006〕22号，以下简称“中央《决定》”）提出的重要工作措施，是人口计生系统融入社会主义新农村建设进程的重要切入点。“十一五”时期，各级人口计生部门积极参与社会主义新农村建设，大力开展多种形式的新农村新家庭创建活动，在提高农村人口计生工作水平、促进农民群众观念更新、推动生殖健康和文明富裕等方面起到了积极作用，基本形成了符合地方实际、具有实践特色的统筹解决农村人口问题长效工作机制。
　　“十二五”时期，人口因素在国家经济社会发展中的基础性地位和作用进一步凸显，加快转变经济发展方式、促进经济社会又好又快发展，对农村人口和计划生育工作提出了更高要求。同时，我国农村人口发展形势复杂，农村人口比重较大，稳定低生育水平和提高人口素质任务较重，出生人口性别比持续偏高，养老保障机制尚不健全，人口流动给农村社会带来的诸多问题还未得到有效解决，农村人口和计划生育工作面临新的机遇和挑战。
　　全面深入实施新农村新家庭计划，就是在社会主义新农村建设总体进程中，充分发挥人口计生公共服务职能和管理网络优势，以农民群众民生为根本，以农民家庭需求为导向，通过宣传倡导、优质服务、利益导向、关怀关爱和民主管理，推动农村家庭特别是计划生育家庭的生育文明、健康促进、权利保障、能力发展和素质提升，努力将广大农村家庭建设成为“计划生育、优生优育、文明富裕、身心健康、和谐幸福”的新家庭，促进社会主义新农村建设，为全面做好人口工作、促进人口长期均衡发展奠定坚实基础。各级人口计生部门要从贯彻落实科学发展观、构建和谐社会的高度，充分认识全面深入实施新农村新家庭计划的重要性和紧迫性，将全面实施新农村新家庭计划摆上工作日程，加大措施力度，把农村人口计生工作提高到新水平。

　　**二、**全面深入实施新农村新家庭计划的指导思想、总体目标和基本原则
　　（一）指导思想。
　　以邓小平理论和“三个代表”重要思想为指导，深入贯彻落实科学发展观，围绕中央《决定》和“十二五”规划要求，把全面深入实施新农村新家庭计划纳入社会主义新农村建设总体规划，充分发挥人口计生公共服务网络优势，以农村家庭为主体，以群众需求为导向，促进和提高农村群众生殖健康和生活质量，全面做好农村人口工作，推动社会主义新农村建设进程。
　　（二）总体目标。
　　通过全面深入实施新农村新家庭计划，使全国农村家庭人口文化繁荣发展，计划生育、生殖健康、家庭保健新知识新观念传播渠道畅通，群众实行计划生育更加自觉；男女平等观念蔚然成风，出生人口性别比偏高势头得到有效遏制并逐步趋向自然平衡；优生优育、生殖健康意识和能力明显增强，出生人口素质和生殖健康水平稳步提高；计划生育家庭合法权益得到保障，农村人口计生社会环境进一步优化；家庭美德、社会公德逐步树立，陈风陋俗逐渐破除，农村和谐，家庭和美，邻里和睦，农村人口计生事业与社会主义新农村建设同步推进。到“十二五”期末，在达到社会主义新农村建设标准的行政村中，70%左右的家庭达到“计划生育、优生优育、文明富裕、身心健康、和谐幸福”的新家庭建设基本标准。
　　（三）基本原则。
　　坚持以人为本。把农民家庭特别是农村计划生育家庭的需求满足、权益保障和能力发展作为出发点和落脚点，更加注重利益引导，更加注重服务关怀，更加注重宣传倡导，使新农村新家庭计划成为农村人口计生工作的民心工程。
　　坚持统筹发展。将全面深入实施新农村新家庭计划纳入新农村建设的总体规划，坚持资源共享、协调推进，在农村人口计生阵地建设、设施配备、惠民政策等各方面，做到统筹、整合、融入、共享。
　　坚持因地制宜。根据各地经济社会发展状况、文化习俗及人口计生工作基础差异，坚持实事求是、量力而行、因地制宜、分类指导，形成新农村新家庭计划的不同发展模式与特色品牌。
　　坚持务求实效。以有利于促进农民及其家庭全面发展、有利于促进社会主义新农村建设、有利于促进农村人口计生工作健康发展为标准，科学规划、分步实施、示范带动、有效推进，力争取得明显的社会效益。

　　**三、**全面深入实施新农村新家庭计划的主要任务
　　（一）推进宣传教育进农家，倡导婚育新风。
　　畅通进家入户宣传渠道。大力传播科学、文明、进步的婚育观念，普及人口计生政策法律法规和生殖健康、优生优育知识。免费发放反映新观念、新风尚、新知识的文图音像宣传品，使每个育龄群众家庭每年至少获得1份适宜宣传品。把握新一代农民的特点和需求，把人口计生法律法规、知识宣传纳入农村文化信息资源系统建设中，利用电子设备、通讯设施和网络技术开展入户到人的宣传。在搞好人口计生宣传培训的同时，充分利用农村党员干部现代远程教育系统、教育扶贫远程教学站等，组织农民群众接受农业技术、致富信息、生活知识、健康卫生等方面的教育培训，提高综合素质，培育新型农民。
　　发展农村家庭人口文化事业。积极争取党委、政府，协调相关部门，把农村家庭人口文化建设纳入新农村文化建设总体布局，提倡人口计生宣传文化场所、设施、设备等与新农村建设综合配套设施共享共用，避免重复建设。结合当地乡风民俗，组织开展群众性文化文艺活动，寓教于乐，吸引群众广泛参与。围绕家庭人口文化的新内涵和新要求，联合有关部门和社会团体，开展多种形式的精神文明创建活动，树立婚育新风尚，建设和谐新农村，营造美满新家庭。
　　（二）推进生殖保健进农家，提供优质服务。
　　做好避孕节育服务。普及避孕节育知识，指导农村育龄群众知情、自主地选择安全、有效、适宜的避孕节育方法，全面落实免费计划生育基本技术服务制度，提高避孕节育有效率。
　　做好生殖健康服务。将依法管理和优质服务相结合，实施生殖健康促进计划，满足育龄群众基本的计划生育、生殖健康服务要求，降低农村非意愿妊娠和流引产发生率。指导育龄妇女积极预防和治疗生殖疾病，提高群众生殖健康水平。
　　做好个性化服务。根据人的生命周期各阶段需求，开展计划生育、优生优育、青少年性健康和中老年期健康等宣传咨询。引导农民群众积极参加孕前优生健康检查，提高出生人口素质。鼓励有条件的地方开展健康教育、健康检查和健康咨询等家庭保健服务，提高农民健康水平。
　　（三）推进奖励优惠进农家，体现利益导向。
　　完善政策推动机制。把健全农村计划生育家庭奖励优惠政策纳入社会主义新农村建设政策配套体系，协调相关部门在制定和落实各项惠农政策时，对农村计划生育家庭予以优先优惠；进一步完善计划生育利益导向政策，使农村计划生育家庭优先分享改革发展成果。
　　保障农村计划生育家庭利益。落实农村计划生育家庭免费、奖励、扶助等制度，探索多种形式的救助、救济、补助等惠民利民政策，提倡农村利用现有资源，对计划生育家庭在普惠政策基础上给予倾斜，在集体利益分配中体现优惠。实施少生快富工程，为农村计划生育家庭提供资金、技术、信息服务，增进计划生育家庭发展能力，促进其能致富、先致富、快致富。
　　（四）推进关爱行动进农家，建设和谐家园。
　　关爱女孩家庭。发挥舆论导向和科普教育的作用，营造有利于农村女孩健康成长和妇女发展的良好舆论氛围。在制定落实新农村普惠政策时，体现计划生育女孩家庭同等优先地位。修订完善村规民约，保障计划生育女孩家庭合法权益，采取多种形式为农村女孩及其家庭提供社会服务和救助。综合治理出生人口性别比偏高问题，依法打击侵害妇女、女孩合法权益的违法犯罪行为。
　　关爱老年家庭。全面落实农村计划生育家庭奖励扶助制度。建立农村养老保障制度过程中优先考虑农村计划生育家庭的利益。引导农民群众大力发扬尊老敬老的良好社会风尚，发挥家庭养老保障的基本功能，积极应对农村人口老龄化问题。
　　关爱特别家庭。对人口流动造成的农村留守家庭、空巢家庭，积极开展生产生活帮扶和身心健康关怀活动，引导家庭成员协助做好外出流动人员计划生育服务管理工作。进一步完善和落实计划生育家庭特别扶助制度，重视解决计划生育特殊困难家庭最直接、最紧迫、最基本的困难和问题。
　　（五）推进村民自治进农家，促进民主管理。
　　维护农民合法权益。开展人口和计划生育法律法规宣传教育活动，组织引导群众知法懂法，履行法定义务，维护合法权益。组织引导农民群众自我教育、自我管理、自我服务，参与民主选举、民主决策、民主管理、民主监督。畅通农民计划生育诉求和维权渠道，简化办事程序，保障合法利益。
　　加强计生民主管理。把人口计生相关内容纳入村民自治章程或村规民约，修订村规民约中性别不平等的资源分配制度等内容，加强村级制度建设，使之成为群众的自觉行为规范。通过多种形式听取群众意见，实行村务公开，接受群众监督，维护群众的人口计生知情权、参与权和监督权。

　　**四、**全面深入实施新农村新家庭计划的保障措施
　　（一）加强领导，科学规划。
　　各级人口计生部门要充分认识全面深入实施新农村新家庭计划的意义，列入重要议事日程，研究制定实施方案。各地要科学规划、稳步推进，采取先试点、后推广的方式加快新农村新家庭计划的实施，及时总结经验，研究解决问题，扎实做好工作。
　　（二）部门协作，形成合力。
　　新农村新家庭计划涉及农村工作诸多方面，要积极争取党委、政府的领导和支持，协调相关部门和社会各界参与配合，整合农村各种资源，共同推进活动的深入开展。人口计生系统要发挥主导作用，不断提高基层人口计生队伍业务能力和服务水平。发挥计划生育协会等群团组织的作用，积极开展计划生育村民自治。
　　（三）因地制宜，分类指导。
　　要充分借鉴国内外项目经验，结合自身实际，创造性地开展工作，不断探索新思路，培育新典型，使新农村新家庭计划更加贴近基层实际，满足群众需求，促进新农村建设。国家人口计生委每年将组织开展调查评估和经验交流，推动工作科学发展。
　　（四）加大投入，提供保障。
　　各级人口计生部门要切实加大对新农村新家庭计划的投入力度，指定专人负责，设立专项资金，确保工作取得积极成效。充分调动专家学者的积极性，为工作发展提供科学指导和智力支持。

　　二○一一年四月一日

© 北大法宝：（[www.pkulaw.com](https://www.pkulaw.com/)) 专业提供法律信息、法学知识和法律软件领域各类解决方案。北大法宝为您提供丰富的参考资料，正式引用法规条文时请与标准文本核对。
欢迎查看所有[产品和服务](https://www.pkulaw.com/helps/2.html)。法宝快讯：[如何快速找到您需要的检索结果？](https://www.pkulaw.com/helps/70.html)[法宝](https://www.pkulaw.com/helps/69.html)V6有何新特色？

扫描二维码阅读原文

**原文链接：**<https://www.pkulaw.com/chl/aa7837b77aaa0789bdfb.html>
